# Husband's intention to support during pregnancy for the use of maternity waiting home in Jimma Zone, Southwest, Ethiopia: a community-based cross-sectional study

**DOI:** 10.3389/fgwh.2024.1342687

**Published:** 2024-06-17

**Authors:** Mamusha Aman, Adisu Bekele, Fira Abamecha, Yohannes Kebede Lemu, Abraham Tamirat Gizaw

**Affiliations:** ^1^ Department of Health, Behavior, and Society, Faculty of Public Health, Institute of Health, Jimma University, Jimma, Ethiopia; ^2^ Mana District Health Department, Oromia, Ethiopia

**Keywords:** husband, maternity waiting home, supporting pregnant women, MWH, husband intention

## Abstract

**Background:**

Husbands are the primary decision-makers about the place of childbirth. Lack of husbands' support for maternal health care is associated with low maternal waiting home utilization and less is known about the husbands' intention to support their wife's use of maternal waiting homes (MWHs) and underlying beliefs in Ethiopia. This community-based cross-sectional survey aimed to study husbands' intention to support during pregnancy through the use of maternity waiting homes in Jimma Zone, Southwest Ethiopia.

**Method:**

A cross-sectional study was conducted among 396 randomly selected husbands whose wives were pregnant. Interviewer-administered, a structured questionnaire developed based on the Theory of Planned Behavior (TPB) was used to collect the data. Multivariable logistic regression analyses were used to examine the association between behavioral intention and constructs of the theory of planned behavior.

**Results:**

Of the 396 husbands who took part in the study, 42.7% intend to support their partner's use of a maternity waiting home. Intention to support a wife to use a maternity waiting home was associated with subjective norm [AOR = 1.303, 95% CI (1.054, 1.611)] and perceived behavioral control [AOR = 1.446, 95% CI (1.234, 1.695)]. Among the control beliefs, “having childcare”; “having a person who stays with a wife at a maternity waiting home”; and “availability of quality service provided to a wife in the maternity waiting home” significantly separated intenders and non-intenders.

**Conclusion:**

The findings suggest that husbands who perceived more social pressure and felt in control of barriers were more likely to intend to support their partner in using a maternity waiting home. Intervention should focus on underlying normative and control beliefs to improve the husband's intention.

## Background

Ethiopia has adopted a variety of policies, including maternal waiting homes (MWH), to minimize maternal mortality (1). In MWH, community-based shelters housed by the health center or hospital, pregnant women remain until birth in the last trimester. It is an integral component of a holistic package aimed at overcoming the second delay (2). In recent years, the government of Ethiopia has established a range of health facilities to increase access to health care for people living in rural areas (3). While there is a difference between countries, more than half of the health facilities have MWH (4).

MWHs play an important role in reducing preventable maternal mortality and negative pregnancy outcomes (5, 6). Studies in Ethiopia also found MWH use accounted for 92% (0.04–0.19) reduction in maternal mortality, 83% (0.05–0.58) (9.90–11.4) reduction of stillbirth, and was associated with a lower rate of direct obstetric complications (5, 6). Despite its effectiveness in minimizing maternal mortality and adverse pregnancy effects, the use of MWH in Ethiopia is very limited. In a recent survey, it was found that only 7% of women used MWH (7). Studies also recorded that the shortage of service and transport infrastructure had a negative effect on MWH use (8–12). Many studies have identified individual and community-related influences; such as lack of awareness and limited husband supports in MWH (13, 14).

In a patriarchal society, the position of the husband goes beyond offering financial assistance to the family; they are the ones who determine where to give birth and seek care (15–17). Husbands' involvement in helping women in reproductive health care has been linked with improved maternal health benefits and better birth outcomes (18–20). The participation of husbands in maternal and infant health care has been advocated as a method to increase the use of maternal health services (21). However, studies in Ethiopia (22, 23) and other sub-Saharan African countries such as Ghana (24) Nigeria (17) and Uganda (25) have shown that husbands participate in maternal health care by assisting their wives in the use of maternal health facilities such as facility-based childbirth, but this participation remains limited.

Studies of factors that affect the engagement of husbands in MWHs have identified various factors that range from context to context. For example, studies in Liberia recorded a lack of food and respectful treatment among MWHs (9, 10). On the other hand, a study in Northern Sierra Leone showed that social and cultural traditions, lack of interest, engagement in other events, distance, and the nature of health facilities are obstacles to the participation of men (8).

Similarly, qualitative research with husbands in Zambia recorded that poor MWH conditions (e.g., lack of room, bedding, water, and sanitation facilities) and the lack of anyone to look after children at home were affected by husbands' decisions to let their wives use MWHs (26). The most prominent quotes from this study are as follows: “Empowering mothers, men, and families to recognize maternal health threats during pregnancy and to take responsibility for designing and enforcing effective responses to them” is a key measure implemented by the Ethiopian Federal Ministry of Health to reduce maternal deaths (27).

Husbands are the main decision-makers in a maternity place, with or without the presence of a mother. The wife hardly gives birth at health facilities without the assistance of her husband (15, 28, 29). Help for maternal health care by the husband may take various forms, such as enabling or promoting the use of resources, providing financial and emotional support, accompanying facilities, and joint decision-making on the place of delivery (30). The lack of support or consent for a husband was a cause for home delivery in recent studies in Ethiopia (16, 28, 29). These results underscored the need to examine the reasons behind the husbands' behavioral intention to plan intervention. However, in Ethiopia, no studies have investigated the intention of spouses, predictors of intent, and attitudes relevant to endorsing the use of MWHs, a portal to facility-based childbirth (22, 23).

Understanding the behavioral intention and the underlying causes will inform action aimed at increasing maternal healthcare use through the involvement of the husband (31). According to the Theory of Planned Behavior (TPB), intent (readiness to execute a given behavior) is the key determinant of behavior. Whereas the intention is the role of the behavioral attitude, the subjective norm, and the perceived behavioral influence. Thus, the desire to conduct a safe action is based on a constructive appraisal of the action, a belief that the referents approve of the action, and a sense of the power of the obstacles and the facilitators of the action (31, 32).

TPB assumes that attitude, subjective norm, and perceived behavioral control are determined by silent beliefs. Attitude is determined by behavioral belief and evaluation of outcome. Normative belief and motivation to comply with referents' expectations determine the subjective norm. Similarly, perceived behavioral control determines control beliefs (beliefs about factors that facilitate or hinder action) and the power of these factors to facilitate or impede acting. The theory proposes that these beliefs influence intention through their corresponding construct (32).

TPB enables the identification of predictors of behavioral intention and underlying beliefs that inform interventions to change or modify behavior. Thus, we undertook this study using the theory of planned behavior as a framework to explore predictors of husbands' intention to support wives' use of MWH and to identify underlying beliefs. TPB allows the recognition of behavioral goal predictors and core values that inform action to alter or improve behavior. We have therefore conducted this analysis using the TPB as a basis for examining the predictors of the husband's role in increasing the intention and the uptake of MWHs among pregnant women.

## Methods

### Study setting and design

A community-based cross-sectional study was undertaken in three randomly selected districts of the Jimma zone (Manna, Kersa, and Seka Chekorsa). The Jimma Zone is located in the western part of Ethiopia. The zone had 121 public health centers, 5 public hospitals, and 512 public health posts at the time of the study. We conducted the study from February 20 through 30, 2019.

### Study populations, sample size, and sampling procedure

The study populations were husbands whose wives were pregnant and living in the selected districts during the study period.

The sample size of 429 was calculated by Epi Info™ 7.1.5.2 based on the size of the population of husbands who have a pregnant wife (800), a 95% confidence level, the proportion of husbands who intend to support their wives using MWH (50%), a 0.05% margin of error, a design effect of 1.5 to account for the heterogeneity between clusters, and 10% non-response.

Three districts were selected randomly from the districts with MWH coverage of 50% and above, i.e., the percentage of health centers accompanied by MWH as contingent maternity care provision centers. During the study period, out of 21 districts in the zone, 10 had 50% or higher coverage. We obtained a list of husbands with pregnant wives from the catchment areas with functional MWHs in the chosen districts through the assistance of health extension workers. The sample size for each area was determined based on the proportion of the eligible husband population within that specific location. Utilizing this compiled list as a sampling frame, the study participants were selected using a simple random sampling technique to ensure a fair representation of the population in the study.

### Instruments and measurements

We developed the instrument based on the TPB manual's suggestion (31): we conducted a qualitative elicitation study in the Gomma district among 20 husbands who had a pregnant wife to explore beliefs salient to support MWH service utilization. Open-ended questions were used to identify behavioral, normative, and control beliefs held by participants about supporting their partner to use MWH for their current pregnancy. The assessment produced eight, six, and seven major behavioral beliefs, normative, and control beliefs, respectively. Subsequently, the beliefs were used to develop belief-based indicators of intention. Finally, behavioral, normative, and control beliefs were weighted by corresponding values of evaluation of beliefs, motivation to comply, and power of the controls. The direct measure assessed four constructs of TPB: intention assessed through five items [Cronbach's alpha (α) = 0.88]; attitude measured with four items [Cronbach's alpha (α) = 0.89]; subjective norms measured with four items [Cronbach's alpha (α) = 0.83]; perceived behavioral control measured with four items [Cronbach's alpha (α) = 0.72]. All direct measures were finally assessed using five-point bipolar adjectives.

### Data collection

Data were collected using a structured questionnaire administered by an interviewer. The questionnaire was first developed in English. The English version of the questionnaire was translated into Afan Oromo and back-translated into English by an expert who is fluent in both languages to ensure consistency between the two versions. Pretesting of the Afan Oromo version of the tool was done among 21 eligible respondents in another district who share common characteristics with study participants. The questionnaire underwent significant changes to the subjective norm and perceived behavioral control items following the pretesting results. The wording was adjusted for clarity, and the questionnaire was then administered in Afan Oromo after modifying some words.

### Analysis

SPSS (version 20.0) was used to conduct statistical analysis. Descriptive statistics such as frequency, mean, standard deviation, and percentage were used to express categorical variables. The independent variables were age, educational status, occupation, past experience, religion, residence, direct attitude, subjective norm, and perception of control. For categorical variables, we conducted bivariate and multivariate logistic regression analyses to look at the association between independent variables and behavioral intention. A *P*-value of less than 0.05 was considered statistically significant. For continuous variables, we conducted bivariate correlations between direct and indirect measures of TPB using Spearman correlations with *P* < 01 and *P* < 05.

## Results

### The sociodemographic characteristics of the participants

Sociodemographic data for our participants are summarized in Table 1. A total of 429 husbands had been assessed for eligibility, out of which 396 participated in this study, for a response rate of 92%. The mean age of the study participants is 33.07 (±4.76 SD), and approximately two-thirds (68.5%) of the participants are aged 20 to 39 years. The majority of the participants are Muslims (94.2%). Those who had a formal education are 4.5%. It is revealed that most of the respondents involved in the study are farmers (94.2%). Besides, 85.1% of respondents showed their wife had been pregnant before the current pregnancy, and 46% of them reported health facility delivery.

**Table 1:** Husbands characteristics and wife obstetric history Jimma Zone, Ethiopia (*n* = 396), 2019.

Variables	Category	Frequency	Percent
Age in years	20–24	23	5.8
	25–29	80	20.2
	30–34	106	26.8
	35 and above	187	47.2
Ethnicity	Oromo	367	92.7
	Amhara	2	0.5
	Other^a^	27	6.8
Religion	Islam	373	94.2
	Orthodox	20	5.0
	Protestant	3	0.8
Educational status	Illiterate	235	59.3
	Read and write	143	36.1
	Grade 1–8	14	3.5
	Grade 9–12	4	1
Current occupation	Farmer	373	94.2
	Employed	8	2
	Merchant	13	3.3
	Other	2	0.5
Birth history	Yes	337	85.1
	No	59	14.9
Place of birth for a previous pregnancy	At home	184	54
	At health facility	153	46

^a^ Others included Kefa, Dawuro, and Yem.

### Knowledge of maternal waiting home

Almost all (99%) of the respondents heard of maternity waiting homes. Regarding the source of information, family members (66.2%) were the most mentioned source, followed by the media and friends. When asked about the right time to visit MWH for childbirth, only 35.9% reported 14 days before the expected delivery date. More than half (51.3%) of respondents identified seven days before the expected delivery date as the right time to visit MWH. The mean score for knowledge was 1.80 as listed in Table 2.

**Table 2:** Knowledge of MWH of husbands’ in jimma zone, south waste Ethiopia 2019.

Items	Response	Number	%
Have you heard of MWH	Yes	395	99.7
	No	1	0.3
Source of information	Media (Radio/TV)	133	33.6
	family member	262	66.2
	relatives/friends	127	32.1
	HEWs	125	31.6
Time to visit MWH before delivery	3 days	28	7.1
	30 days	23	5.8
	7 days	203	51.3
	14 days	142	35.9
Benefit of MWH	Medical attention	255	64.4
	Bed rest	66	16.7
	Institutional delivery	249	62.9
	Early referral	1	0.3

### MWH utilization experience

In the sampled population, 85.1% reported that their partner or wife had ever given birth, while 14.9% revealed that their partner got pregnant for the first time. In response to questions related to the place where their partner gave birth during the previous pregnancy, 45.4% reported delivery in a health facility, and 54.6% reported home delivery. Sixty-seven percent of participants stated that the distance between their home and MWH is over five kilometres.

### Attitude, subjective norm, perceived behavioral control, and intention

Direct attitude, subjective norm, and perceived behavioral control had a mean score of 16.53 (SD ± 2.12), 14.13 (SD ± 2.23), and 14.23 (SD ± 3.39), respectively. The mean score of intention was 11.76 (SD ± 3.60). Intention is categorized based on the mean score as intended (scored above the mean score) and non-intenders (scored below the median score). Among all respondents, nearly forty-three percent (42.7%) of them intend to provide support (Table 3).

**Table 3:** Descriptive statistics of responses for the indirect and direct measures of TPB, jimma, Ethiopia (*n* = 396), 2019.

Constructs	Items number	Scale range	Scale mean with (SD)	Mean percentage	Cronbach's alpha
Direct Measures					
Attitude	4	4–20	16.53 (2.12)	82.65	0.89
Subjective Norm	4	4–20	14.13 (2.23)	70.65	0.83
Perceived Behavioral Control (PBC)	4	4–20	14.23 (3.39)	71.15	0.71
Intention	5	5–15	11.76 (3.60)	78.4	0. 88
Indirect Measures					
Behavioral belief (BB)	8	8–24	22.70 (2.19)	94.5	0.826
Evaluation of behavioral belief (EB)	8	8–24	21.28 (2.57)	88.6	0.635
Normative belief (NB)	6	6–18	15.65 (2.31)	86.94	0.821
Motivation to comply (MC)	6	6–18	16.43 (2.26)	91.27	0.846
Control belief (CB)	7	7–21	14.95 (3.60)	71.19	0.770
Power of control (PC)	7	7–21	16.18 (1.18)	77.04	0.798

Based on the correlation analysis, all indirect measures positively correlated with their corresponding direct measures, which confirms the validity of the indirect measures. Indirect attitude correlated with direct attitude (*r* = 0.187, *P* < 0.001), a moderate correlation was observed between indirect subjective norm and direct subjective norm (*r* = 0.432, *P* < 0.001), and indirect PBC correlated with direct PBC (*r* = 0.350, *P* < 0.001) (Table 4).

**Table 4:** Pearson correlation between indirect and direct measures of TPB, (*n* = 396), 2019.

Constructs	Intention	DA	DSN	DPBC	IA	ISN	IPBC
Intention	1						
DA	0.06	1					
DSN	0.149**	0.461**	1				
DPBC	0.596**	0.213**	0.299**	1			
IA	0.107*	0.187**	0.352**	0.286**	1	.	
ISN	0.197**	0.208**	0.432**	0.334**	0.665**	1	
IPBC	0.290**	0.095	0.136**	0.350**	0.419**	0.571**	1

DA, direct attitude; DSN, direct subjective norm; DPBC, direct perceived behavior control; IA, indirect attitude; ISN, indirect subjective norm; IPBC, indirect perceived behavior control.

* Correlation significant at the *P*-value 0.05 (2-tailed).

** Correlation significant at the *P*-value 0.01 (2-tailed).

### Factor associated with the intention to support

In the multiple regression analysis, perceived behavioral control (PBC) and subjective norm were positively associated with the husband's intention to support. For each unit increase in perception of control, participants had a 44.6% higher likelihood of intention to support their wives by using maternity waiting homes [AOR = 1.446, 95% CI (1.234, 1.695)]. Likewise, a unit increase in the subjective norm increases the likelihood of intending to support a wife to use a maternity waiting home [AOR = 1.303, 95% CI (1.054, 1.611)] (Table 5).

**Table 5:** Multivariable logistic regression model predicting husband intention to support wife to sue MWH, jimma zone, Ethiopia. (*N* = 396), 2019.

Variables	B	*P*-value	AOR	95% CI	
				Lower	Upper
DSN	0.265	0.014	1.303	1.054	1.611
DA	−0.171	0.088	0.843	0.693	1.026
DPBC	0.369	0.000	1.446	1.234	1.695
Used MWH during the previous pregnancy (Yes)*	0.116	0.749	1.123	0.552	2.282
Distance of MWH from home (<5 kilometer)**	−0.141	0.706	0.868	0.416	1.811
Educational status (formal education)***	0.195	0.587	1.215	0.601	2.457

Maximum standard error = 0.375, Hosmer Lemeshaw (*X*^2^ = 2.63, df = 8; *p* = 0. 955).

Reference groups = *not used MWH, **MWH distance greater than five kilo meters, ***no education.

### Belief identification

We have conducted further analysis to identify beliefs that had the greatest influence on intentions. We did not conduct research on beliefs underlying attitude because attitude was not significantly associated with intention in multiple logistic regression. All normative beliefs were significantly correlated with intention, their correlations varying from only 0.16 to 0.31. Similarly, three control beliefs significantly correlated with intention: presence or absence of a person who looks after children at home (*r* = 0.37, *P* < 0.01); presence or absence of a person who cares for a wife at MWH (*r* = 0.36, *P* < 0.001); and quality of services provided in the MWH (*r* = 0.11, *P* < 0.01) (Table 6).

**Table 6:** Normative and control beliefs correlation with intention, jimma zone, southwest Ethiopia, 2019.

Variables	Point biserial correlation	Mean Belief	
		Intenders	Non-intenders
Normative Belief			
My mother/mother-in-law thinks I should support my wife to use MWH services by staying there for two weeks before childbirth, for the current pregnancy.	0.168**	7.9112	7.1542
My father/father-in-law thinks I should support my wife to use MWH services by staying there for two weeks before childbirth for the current pregnancy	0.169**	7.8166	7.0705
My wife would likely approve of my supporting her to use MWH services for current pregnancy by staying there for two weeks before childbirth	0.177**	8.5385	8.0044
Members of my group support their wife to use MWH services for childbirth by staying there for two weeks before the date of delivery.	0.314**	7.7751	6.2863
Women development army leaders think I should support my wife to use MWH services for current pregnancy by staying there for two weeks before childbirth.	0.286**	7.5917	6.2291
Leader's approval of my effort to support my wife to use MWH services for current pregnancy by staying there for two weeks before childbirth	0.266**	7.5621	6.2379
Control Belief			
Transportation services (vehicles) are available to take my wife to MWH to stay two weeks before birth for services.	0.087	6.9349	6.4273
I expect that my wife will not get enough food service in MWH, if she goes to stay at MWH two weeks before birth for services.	−0.028	4.9763	5.1101
Traditional coffee ceremonies, porridge, or home-like environments are available for pregnant women at MWH.	0.016	6.9349	6.8546
I expect that there will be a person who cares for my children at home when my wife goes to MWH for two weeks before delivery.	0.370**	6.9586	4.7841
I have a person who cares for a wife at MWH if my wife goes to stay at MWH for two weeks before delivery for services.	0.367**	7.1124	4.9780
Health workers who provide services at MWH are not adequate.	−0.017	5.0355	5.1057
0The services provided to the pregnant women in MWH are not satisfactory.	0.113*	5.7219	5.2511

* Correlation significant at the *P*-value 0.05 (2-tailed).

** Correlation significant at the *P*-value 0.01 (2-tailed).

## Discussion

TPB can be utilized to change the husband's behavior and increase support for the utilization of MWHs. The TPB posits that behavioral intentions are influenced by three key factors: attitude toward the behavior, subjective norms, and perceived behavioral control. In this case, changing the husband's behavior to support the utilization of MWH would involve evaluating attitude toward this behavior, the influence of subjective norms, and perceived control over engaging in this supportive behavior.

This study examined psychological factors influencing the intention to support the wife in using MWH among husbands. The findings revealed that subjective norms and perceived behavior control were significantly associated with the intention to support the wife in using MWH. Forty-two percent of respondents reported that they intend to support their wives using MWH for their current pregnancy. In this study, we did not measure behavior, but the intention is assumed to be the immediate predictor of behavior (32, 33); thus, the low involvement of the husband in maternal health care reported in previous studies (22, 23) could be explained by low motivation. Given the high preventable maternal and child mortality in the country, improving husbands' intentions is crucial to reducing maternal mortality by increasing access to and utilization of maternal health services (19, 20).

In line with previous studies (7, 9), the respondents listed “medical attention” and “institutional delivery” when asked about the benefits of MWH. With regards to the time to visit the MWH, 12.8% of respondents did not know the right time. To avoid delays in utilizing MWHs, healthcare professionals must focus on raising awareness about the appropriate time to visit MWHs. This is crucial because an insufficient understanding of the ideal timing for seeking care can result in missed opportunities for receiving MWH services.

The bivariate analyses revealed that attitude towards supporting a partner to use maternal waiting services showed statistically significant associations with intention. However, when controlled for other variables, the association becomes non-significant. The essence of the behavior and study participants may be explained as follows: Traditional gender roles delineate pregnancy and childbirth-related issues for women (17, 34, 35). The tie of target behavior with social values and the collectivist nature of participants' culture may subdue personal feelings (36, 37).

In our study, subjective norms were positively associated with the intention to support the use of MWH by women. This has demonstrated that supporting the wife to use MWH was influenced by perceptions about what the others do and referents' approval of supporting the wife to use MWH. Previous studies also showed that social pressure is one factor that influences male involvement (35, 38). This finding highlights the importance of targeting social norms to improve husbands' intentions and practices related to supporting wives to use MWH. An intervention needs to address beliefs about the approval and practice of referents with factors in a broader social context to change social norms and increase intentions and related behavior (39).

Perception of control was another predictor of intention to support a wife using MWH among husbands. In an analysis aimed at identifying the beliefs that discriminate between intenders and non-intenders, only three control beliefs were found to have significant influence or discriminate between two groups: having someone who takes care of children at home, having a person who cares for a wife in MWH, and quality service at MWH.

Husbands are income earners in the family (35) and they may be busy with activities to support the family economically (25). This could lead them to perceive the absence or presence of a person who looks after children at home and a wife in MWH as a barrier or facilitator. Moreover, belief about the quality of services provided at MWH was mentioned as one factor. This finding is consistent with previous studies that reported a lack of space for men, the absence of health workers, and the lack of amenities as barriers (9, 11).

MWHs have become an important intervention in Ethiopia to improve maternal health outcomes. In addition to the expansion of MWHs, there are ongoing community-level promotions to create demand and engage the community. However, those who have used the services reported several limitations (7). This suggests the government and stakeholders need to improve the quality of services provided in the MWHs, as poor quality of service negatively affects maternal health service utilization (40, 41).

## Strengths and limitations

This study has the strength to note: (1) This study is the first to analyze the role of husbands in increasing MWH uptake; (2) the qualitative salient beliefs were explored and integrated into the beliefs dimensions for the quantitative study; and (3) a standard pretested structured questionnaire was used. However, this study has some potential shortcomings that should be noted: (1) First, only husbands whose wives were pregnant were included in the study. Thus, the results may not be inferred from the general population of male partners; (2) this study used data obtained from self-report that might be subjected to social desirability bias. We tried to minimize these issues by recruiting experienced interviewers, providing a brief overview of the study, and ensuring that their responses were not linked to them in any way. (3) The associations reported in this study were so correlational that we cannot make a causal link. Finally, we did not measure actual behavior and incorporated variables that predict intention, such as past behavior. Therefore, future research may extend this by measuring actual behavior.

## Conclusions

The present research used TPB to determine psychological factors impacting the intention of husbands to support their wives in the utilization of MWH. The findings of the study indicated that the subjective norm and perceived behavioral control were associated with the intention to support MWH utilization.

In a study aimed at defining particular beliefs, all the normative beliefs and three control beliefs, such as having a person who looks after children in the home and a wife at MWH, and the quality of services provided at MWH, were separated between intenders and non-intenders. Ajzen suggests interventions aim to promote change in practice through intention and the need to target underlying beliefs (31, 32). Therefore, to increase the utilization of MWHs, interventions should focus on targeting both normative and control beliefs, particularly through the support of husbands. By addressing societal norms surrounding maternity care that may prevent women from utilizing the services, interventions have a higher likelihood of success in promoting MWH utilization. By involving husbands in the decision-making process and support, women may feel more empowered to access MWHs and ultimately improve their maternal health outcomes.

## Data Availability

The original contributions presented in the study are included in the article/Supplementary Material, further inquiries can be directed to the corresponding author.
